# Effect of Ramadan intermittent fasting on inflammatory markers, disease severity, depression, and quality of life in patients with inflammatory bowel diseases: A prospective cohort study

**DOI:** 10.1186/s12876-022-02272-3

**Published:** 2022-04-24

**Authors:** Mohamed Negm, Ahmed Bahaa, Ahmed Farrag, Rania M. Lithy, Hedy A. Badary, Mahmoud Essam, Shimaa Kamel, Mohamed Sakr, Waleed Abd El Aaty, Mostafa Shamkh, Ahmed Basiony, Ibrahim Dawoud, Hany Shehab

**Affiliations:** 1 Integrated Clinical and Research Centre for Intestinal Disorders (ICRID), Endemic Medicine Department, Faculty of Medicine, Cairo University, Giza, Egypt; 2 Endemic Medicine Department, Faculty of Medicine, Ain Shams University, Cairo, Egypt

**Keywords:** Intermittent fasting, Ramadan fasting, Inflammatory bowel diseases, Ulcerative colitis, Crohn’s disease

## Abstract

**Background:**

Intermittent fasting (IF) during the month of Ramadan is part of the religious rituals of Muslims. The effect of intermittent fasting on disease activity in inflammatory bowel diseases (IBD) is still unknown. This is the first study to assess the effect of IF during Ramadan on inflammatory markers in patients diagnosed with IBD. The effects on clinical disease activity, quality of life, and levels of depression were also assessed.

**Methods:**

Patients diagnosed with ulcerative colitis (UC) or Crohn’s disease (CD) who intended to observe Ramadan fasting were recruited. The following were assessed immediately before and at the end of Ramadan: Serum CRP and stool calprotectin, partial Mayo score, Harvey Bradshaw index (HBI), Simple IBD questionnaire (SIBDQ), and Hamilton depression scale questionnaire.

**Results:**

80 patients diagnosed with IBD were recruited (60 UC, 20 CD). Serum CRP and stool calprotectin did not show a significant change before vs after fasting (median CRP 0.53 vs 0.50, *P* value = 0.27, Calprotectin 163 vs 218 respectively, *P* value = 0.62). The partial Mayo score showed a significant rise after fasting (median 1 before vs 1 after fasting, mean: 1.79 vs 2.33 respectively, *P* value = 0.02). Harvey-Bradshaw index did not show a significant change after fasting (median 4 vs 5, *P* value = 0.4). Multiple linear regression revealed that older age and a higher baseline calprotectin were associated with a higher change in Mayo score after fasting (*P* value = 0.02 and *P* value = 0.01, respectively). No significant change was detected in SIBDQ or Hamilton depression scale scores.

**Conclusions:**

In patients diagnosed with UC, IF during Ramadan was associated with worsening of clinical parameters, the effect was more pronounced in older patients and those with higher baseline calprotectin levels. However, IF during Ramadan was not associated with an adverse effect on objective inflammatory markers (CRP and calprotectin).

## Introduction

Ramadan is the ninth month of the lunar calendar and is considered a Holy month in the beliefs of about 2 billion Muslims all over the world. Fasting throughout the daytime during the month of Ramadan is considered one of the pillars of Islam, and except for pregnant women, the sick, and the elderly, all adult Muslims are expected to fast every day throughout this month. Fasting during Ramadan is one of the intermittent fasting (IF) regimens that mimics several protocols used frequently for weight loss and other health benefits, especially the time-restricted eating (TRE) [1–3] Ramadan intermittent fasting (RIF) entails a complete abstinence from food and liquids from dawn to sunset. Counselling patients with IBD on whether fasting will benefit or harm their bowel condition remains difficult due to the lack of studies on this issue.

The term IF encompasses several regimens of fasting such as alternate day fasting and fast-mimicking diet, yet most commonly it refers to time-restricted eating which usually lasts between 12 to 16 h daily [2]. Intermittent fasting has become a point of interest for researchers as beneficial effects on health have been demonstrated [3–5]. On the other hand, some studies have warned of possible deleterious effects of IF on some health conditions [6, 7]. The ability of IF to reduce systemic inflammation has been of particular interest, when considering RIF in particular, basic research has shown mostly a positive impact on inflammatory and oxidative stress markers, many of which are involved in the pathogenesis of IBD such as IL-1, TNF-alpha, IL-1 beta, and C-reactive protein (CRP) [8, 9]. Clinical studies on RIF in autoimmune disorders such as rheumatoid arthritis (RA) and psoriasis have shown improved activity indices and a reduction in inflammatory markers in both diseases after a period of RIF [10, 11]. Whether this effect will be replicated in inflammatory bowel diseases is still unknown. Only the clinical effect of RIF on patients diagnosed with IBD has been reported in 2 studies; one reported no clinical deterioration while the other reported some improvement in clinical parameters [12, 13]. Two studies are currently underway to assess fasting-mimicking diet and intermittent reduced-calories diet as therapeutic tools in IBD [14, 15].

To our knowledge, no study to date has assessed the effects of RIF on inflammatory markers in IBDs. In this study, we assessed the impact of RIF on serum CRP and faecal calprotectin (fCal), as well as disease activity indices, quality of life, and depression scales in patients diagnosed with IBD.

## Methods

This was a prospective observational cohort study during the lunar month of Ramadan 2021 (1442 Hijri year). The study was performed at 2 tertiary referral centres for IBD in Egypt: The integrated clinical and research centre for intestinal disorders, Cairo University, and the Inflammatory bowel diseases clinic, endemic medicine department, Ain Shams University. All patients who were independently willing to fast during the month of Ramadan this year were assessed for eligibility. Inclusion criteria included: Age ≥ 18 years of age and less than 70 years, on the same drug regimen for the past 12 weeks at least with no plan to change drug regimen within the coming month and known adherence to medications. Exclusion criteria included: any change in medications dosage or type during the previous 12 weeks, history of perforation of the bowel or megacolon, active infection, presence of other systemic diseases (such as diabetes, cardiovascular diseases, or any other disorder that may be aggravated by fasting), elevated serum creatinine above normal, elevated transaminases > 2 × ULN and known alcohol or drug abuse. Patients on immunomodulators, biological therapies, or corticosteroids were not excluded from the study (unless a change in dose within the following month is anticipated).

During the month before Ramadan, all eligible patients from our IBD registries were contacted. No instruction or recommendation to fast was given to any patient. However, patients were advised not to fast if they had experienced previous harmful effects from fasting or if they had any other comorbidities that could be exacerbated by fasting (e.g., uncontrolled diabetes). Ethical committee approval was obtained in both centers (approval numbers: N-27–2021, R 93/2021). After a written informed consent, assessments were done twice at the following time points: the first, during the week immediately before Ramadan, the second, during the last 3 days of Ramadan or 3 days immediately after Ramadan.

Assessments included: inflammatory markers (CRP, fCal), clinical disease activity (partial Mayo score for patients with UC and Harvey Bradshaw index (HBI) for patients with CD), quality of life (short inflammatory bowel disease questionnaire, SIBDQ), and depression levels (Hamilton depression rating scale) [16–19].

### Fasting details

Fasting for Muslims during the month of Ramadan entails complete abstinence from food and liquids intake, smoking, and sexual activities from dawn to sunset. In Egypt, this year at the start of Ramadan sunrise was at 3:59 while the sunset was at 18:23, i.e., a duration of just more than 14 h of complete fasting, by the end of Ramadan this reached 15 h of fasting a day due to the lengthening of daytime. At night, between sunset and dawn, there is no restriction to any type of the usual *Halal* food or liquids intake.

### Assessment of disease activity

Quantitative fCal levels were assessed by ELISA technique (ORG 580 kit, Orgentec diagnostika GmbH). CRP level assessment was done using nephelometry methodology (mispa-i2, Agappe Diagnostics GmbH). Blood sampling included a 5 cc whole blood sample taken in the morning after an 8 h fast, samples taken during Ramadan were shifted to noon only if a late sohour meal was taken and thus ensure an 8 h fast. Clinical disease activity was assessed by either the HBI in cases of CD or the partial Mayo score in cases of UC.

### Quality of life and depression questionnaires

The SIBDQ consists of ten questions from the Inflammatory Bowel Disease Questionnaire (IBDQ) that record disease-specific quality-of-life on bowel-, systemic-, emotional- and social subscales [19]. An overall score can be calculated and ranges from 10 to 70. This self-administered questionnaire was translated according to Behling and Law's technique [20]. We appointed three translators with excellent proficiency in both Arabic and English languages. The first translator translated the original version into Arabic. The second translator back-translated the translated version into English. The third translator compared the original and back-translated versions and prepared the final draft with minor modifications to adjust to Egyptian patients. The assessment of depression was performed by the Hamilton depression score, this has already been translated and validated in Arabic [21].

### Statistical analysis

Wilcoxon Signed Ranks test was used to compare different parameters before and after Ramadan fasting, change in different scores and lab investigations (CRP and fCal) was calculated by subtracting the value before fasting from the value after fasting, then multiple linear regression analysis was done to measure the independent effect of different parameters on these changes. Analysis of data was done using SPSS program version 25. Quantitative variables were presented as median and Interquartile range (IQR). Qualitative variables were presented as count and percentage. *P* value < 0.05 was considered statistically significant.

Sample size: To our knowledge, this was the first study to assess the effect of intermittent/Ramadan fasting on inflammatory markers in inflammatory bowel diseases. In this respect, this is considered a pilot study, and thus no power-based sample size estimate was possible or appropriate. We chose to include all our clinic patients meeting the inclusion criteria with a minimum of 50 subjects for our primary outcome measure: CRP and fCal levels (according to the estimate by Sim and Lewis, the minimum recommended number of subjects for a pilot clinical study is 50) [22].

## Results

Out of 88 eligible patients contacted, 80 patients (91%) were intending to fast during Ramadan and were included: 60 UC and 20 CD, median age 32 years (range 18–64 years), 41 males & 39 females (Table 1). Forty patients (50%) were on biologics (33 Infliximab, 4 adalimumab, 2 ustekinumab, 1 vedolizumab), 57 (71%) were on immunomodulators (55 azathioprine, 1 mycophenolate mofetil, 1 methotrexate), 51 (64%) on oral mesalamine and 15 (19%) were on low-dose oral steroids (< 10 mg prednisolone). The median partial Mayo score was 1 (range 0–6), median HBI was 5 (range 0–15). During the study, one patient had an iatrogenic perforation during a routine screening colonoscopy and was thus excluded from the study. Two UC patients stopped fasting due to deterioration of their condition after 20 and 22 days, their assessments were performed at the time of stopping and were not excluded. Both patients had resolution of their symptoms by oral prednisolone 20 mg. No other patient required dose adjustment or change in medication during the study period. No hospitalizations or ER visits were recorded during the study period.

**Table 1:** Baseline demographics

Number	80
Age*	32 (18–64)
Sex	Males 41 (51), females 39 (49)
Smoking	4 (5), all CD
Disease type	UC 60 (75) CD 20 (25)
Time since diagnosis (years)*	3 (1–15)
*Disease location*	
UC	
Left-sided Pancolitis Ileocolonic Isolated ileal Proximal small bowel	31 (52) 29 (48) 14 (70) 5 (25) 1 (5)
Disease Phenotype (CD)	
Stenosing/penetrating Inflammatory	12 (60) 8 (40)
*Medications*	
Biologics Immunomodulators Steroids Oral Mesalamine *Number of current medications* 0 1 2 3 4	40 (50) 57 (71) 15 (19) 51 (64) 3 (4) 16 (20) 38 (47) 20 (25) 3 (4)
*Disease activity*	
Partial Mayo score* HBI*	1 (0–6) 5 (0–15)

Number (percent)

^*^Median (range)

Serum CRP did not show a significant change before vs after fasting (median 0.53 vs. 0.50 mg/dl respectively, *P* value = 0.27) (Table 2). fCal did not differ significantly before vs after fasting (median 163 vs. 218 mcg/g respectively, *P* value = 0.62). In UC patients, the median partial Mayo score showed a statistically significant rise after fasting (1 before vs. 1 after, *P* value = 0.02, mean: 1.79 vs 2.33 respectively), while in CD patients the median HBI did not differ significantly before vs. after fasting (4 vs. 5 respectively, *P* value = 0.41). SIBDQ score showed no significant difference before vs after fasting (median 43 vs. 40 respectively, p *P* value = 0.12). Hamilton depression scale score was also similar before and after fasting (median 18 v. 18 respectively, *P* value = 0.81) as shown in Table 2.

**Table 2:** Changes in inflammatory markers, disease activity indices and quality of life scores between pre-and post-Ramadan IF

	Before fasting		After fasting		*P* value
	Median (Mean)	IQR	Median (Mean)	IQR	
Serum CRP* All UC CD	0.53 (1.32) 0.5 (1.17) 0.98 (1.80)	0.18–1.56 0.12–1.56 0.40–1.70	0.50 (1.74) 0.48 (1.85) 0.81 (1.40)	0.15–1.22 0.15–1.40 0.27–1.19	0.27 0.47 0.67
Stool calprotectin** All UC Crohn’s	163 (276) 176 (295) 152 (228)	35–418 50–436 28–338	218 (323) 218 (358) 206 (230)	63–426 124–613 52–383	0.62 0.34 0.73
Partial Mayo score	1 (1.8)	0–3	1 (2.3)	0–5	0.02
Harvey-Bradshaw index	4 (4.9)	3–5	5 (5.4)	2–7	0.41
SIBDQ	43 (41.4)	29–54	40 (39.8)	30–49	0.12
Hamilton depression scale	18 (16.6)	8–23	18 (16.9)	10–23	0.81

CRP: C-reactive protein, IQR: Interquartile range, UC: Ulcerative colitis, SIBDQ: Simple inflammatory bowel diseases questionnaire

^*^ 65 patients had CRP tested before and after fasting

^**^ 51 patients had stool calprotectin tested before and after fasting

A subgroup analysis was performed to assess factors associated with the significant rise in partial Mayo score (Table 3). The following groups showed a significant association with worsening partial Mayo score after fasting: older patients, males, those with a lower baseline Mayo score, and those with higher baseline fCal levels (Table 3). However, multiple linear regression analysis revealed that only higher age and higher baseline fCal were significantly associated with a rise in partial Mayo score after fasting (*P* value = 0.02 and *P* value = 0.01, respectively) (Table 4).

**Table 3:** Variables associated with change in partial Mayo score

	Partial Mayo before fasting		Partial Mayo after fasting		*P* value
	Median (Mean)	IQR	Median (Mean)	IQR	
Male	1 (1.5)	0–2	2 (2.4)	0–5	0.02
Female	2 (2.1)	0–3	1 (2.3)	0–5	0.34
Age < 30	1 (1.6)	0–2	1 (1.9)	0–3	0.95
Age ≥ 30	1 (1.8)	0–3	3 (2.7)	1–5	0.01
On Biologics	1 (2.4)	1–5	1 (2.8)	0–6	0.10
Not on Biologics	1 (1.3)	0–2	1 (2.0)	0–3	0.07
Baseline Mayo ≤ 1	1 (0.5)	0–1	0 (1.2)	0–1	0.03
Baseline Mayo > 1	4 (3.8)	2–5	5 (4.1)	2–6	0.29
Baseline stool Calprotectin < 200	1 (1.1)	0–1	1 (1.3)	0–2	0.54
Baseline stool Calprotectin ≥ 200	2 (2.3)	0–4	5 (3.5)	1–6	0.05

**Table 4:** Multiple linear regression analysis for factors associated with higher change in Mayo score

	Unstandardized coefficients		Standardized coefficients	*P* value	95% confidence interval for B	
	B	Std. error	Beta		Lower limit	Upper limit
Age	0.814	0.335	0.729	0.02	0.135	1.494
Sex	− .719	.386	− .635	.07	− 1.501	.063
Biologics	− .316	.566	− .108	.57	− 1.462	.830
Baseline Mayo score	− .184	.177	− .236	.30	− .542	.175
Baseline stool calprotectin	.003	.001	.625	0.01	.001	.005

## Discussion

IBD was typically considered a “Western” disease, seldom being encountered in Eastern countries with a higher Muslim population. The situation is changing, however, with more and more eastern, Muslim predominant countries showing a rising prevalence of IBD over the last 2 decades [23]. This translated to a pressing need to know and inform these patients whether fasting during the month of Ramadan could benefit or harm their condition.

The results of numerous preclinical studies favour the idea that fasting may be able to shift the immune environment from pro- to anti-inflammatory and thus have a beneficial role in autoimmune disorders [24]. An important concept in favour of the anti-inflammatory effect of fasting is the shift in immune cell behaviour according to nutrient availability and abundance [25–27]. The mechanistic target of rapamycin (mTOR) for example, an intracellular energy sensor, increases TH1 and TH17 differentiation in the case of an abundance of calories, whilst decreased activation (with caloric restriction) leads to Treg cell differentiation, potentially creating a more anti-inflammatory environment [28]. In multiple sclerosis, 3-day fasting-mimicking diet in mice was found to induce apoptosis of autoimmune Th1 and Th17 cells and enhanced the expansion of the Treg cell population [29]. Intestinal dysbiosis in IBD is characterized by low microbial diversity, decreased *Bifidobacterium spp, Lactobacillus spp,* and *Faecalibacterium prausnitzii,* and increased pathobionts such as invasive E. coli and C.diff resulting in decreased butyrate production [30–35]. Butyrate has powerful anti-inflammatory properties promoting Treg cell proliferation and improving intestinal barrier function [25]. Fasting has been found to increase *Faecalibacterium prausnitzii* and *Akkermansia mucinophilia*, both have been deemed to characterize an anti-inflammatory intestinal environment [24]. Despite the results of these studies favouring the effect of fasting in IBD, immune suppression as a result of prolonged fasting may in theory depress the protective response to pathogens, some which could potentially be implicated in the augmentation of inflammation in IBD. Clinical studies have shown a positive effect of fasting on body weight and adiposity, cardiovascular health, asthma, liver enzymes, and multiple sclerosis [8, 36–42]. The benefit of fasting in autoimmune disorders such as RA and psoriasis has been demonstrated in clinical controlled trials, sparking enthusiasm on whether these results will be mirrored in other autoimmune disorders such as IBD [10, 11]. Interestingly, a study has shown that RIF in obese individuals (not diagnosed with IBD) decreased proinflammatory adipokines such as TNF-α and IL6 (known to have a role in IBD), and promoted anti-inflammatory IL10 known to have a protective role in the intestinal mucosa [8]. However, a recent study on fasting in hospitalized patients with IBD (a form of fasting quite different from RIF) has shown no benefit compared to enteral nutrition [43].

Our results showed a statistically significant rise in partial Mayo score after fasting throughout the month of Ramadan. Calprotectin also showed a rise yet it did not demonstrate a statistical significance, nevertheless, this rise is supportive of the finding that the Mayo score did rise after fasting. Two UC patients had to stop fasting due to the deterioration of their condition while fasting. To our knowledge, two previous studies assessed the effect of RIF on patients with IBD, they did not assess CRP or calprotectin [12, 13]. One of the studies showed a reduction in UC activity index after fasting, however, in this study only 5 patients fasted throughout the month, a number too small to draw any solid conclusion [12]. The other study by Elmountassir et al. conducted on 100 CD patients concluded that fasting was well tolerated by 94% of patients, but no assessment of disease activity indices was performed [13]. Our results are also in contradiction to what was previously reported in rheumatologic diseases such as RA and psoriasis, where some studies have shown an improvement in activity indices as well as a reduction in CRP [10, 11]. One pilot study in systemic lupus erythematosus (SLE), however, showed no effect on disease activity but a significant rise in Anti-dsDNA [44]. IBD shares similar pathophysiological sequences with several other autoimmune disorders and thus treatment is usually very similar. Major differences, however, do exist and can reflect greatly on the clinical management of such patients, e.g. anti-IL17 medications used to treat RA have a deleterious effect on disease activity in IBD [45]. In addition, the fact that IBD is predominantly a pathology of the intestines, makes it vulnerable to different effects of fasting other than those to which rheumatological disorders are exposed. Therefore, it remains plausible that the effects of fasting could be conflicting between IBD and other autoimmune disorders.

It has to be taken into account that RIF includes other factors that could influence disease activity. The timing of oral medications is changed during Ramadan due to the inability to take any medications during the daytime. Many patients who usually take medications twice a day are forced to take them either once a day or with a much smaller interval in between, this may affect compliance to taking the medications with several doses possibly being missed altogether. Sleeping patterns are also usually changed during the month of Ramadan with a possible effect on circadian rhythm [46–49]. Circadian cycle proteins and hormones have a direct effect on the inflammatory response and have shown pro- or anti-inflammatory effects in animal models of autoimmune diseases [49]. These peculiarities of Ramadan fasting mean that it remains important not to apply our results to all types of intermittent fasting.

Our study did not show a statistically recognizable effect on inflammatory markers (CRP and stool calprotectin), despite the numbers showing a trend for a rise. It can be argued that a larger sample size is required to demonstrate this difference. A systematic review assessing the effect of RIF on inflammatory markers in healthy adults concluded that minimal reductions in Il-1 and CRP and small reductions in Il-6 and TNF-α were noted after RIF [50]. Whether these small reductions are related only to the weight loss achieved by RIF is yet to be concluded [50]. The largest meta-analysis to date on the effects of intermittent fasting (other than RIF) on inflammatory markers (in non-IBD patients) has shown a reduction in CRP after fasting, predominantly in obese individuals and importantly, those who follow intermittent fasting for more than 8 weeks, a condition not met in the one-month Ramadan fasting [51]. Notably, this large study also demonstrated no effect on IL-6 and TNF-α, pivotal cytokines in the pathogenesis of IBD [52]. These facts could partially explain why RIF did not demonstrate an improvement in inflammatory markers in IBD patients.

Our multiple logistic regression revealed that older patients and those with higher baseline fCal are more prone to deterioration in their partial Mayo score. A higher baseline fCal is known to be associated with a higher risk of clinical relapse in asymptomatic IBD patients [53]. It is thus rational that those with a higher baseline fCal will be the most prone to clinical deterioration when an inciting factor (fasting) is present. Older patients are more prone to negative effects of fasting such as dehydration and hypoglycaemia, the symptoms of which might raise the “physician global assessment” part of the partial Mayo score [54]. Elderly patients also more commonly have comorbidities and have polypharmacy problems that are compounded during the month of Ramadan. Whether age independently is a factor associated with disease relapse during fasting requires larger studies for confirmation, but for now, it seems sensible to inform elderly patients with UC about the possible deleterious effects of fasting and keep those who do fast on a strict follow-up.

The SIBDQ score showed no significant change after fasting. This could lead to the conclusion that if fasting is associated with a negative effect on IBD, then it does not seem to be significant enough to affect the quality of life of patients related to the disease, this needs to be confirmed however by a study with a larger number of patients. SIBDQ also assesses symptoms within the previous 2 weeks, it might thus be less sensitive to changes that occur within a short period such as a month, in comparison to Mayo score which assesses symptoms in the previous 3 days. We assessed the Hamilton depression scale before and after fasting to negate any possibility that any change in activity indices would be secondary to a change in mood and psychiatric status of the patients. Our results show no significant change in the depression index after fasting. Several other studies on healthy volunteers and different medical conditions have shown contradicting results on the effect of fasting on depression and mood disorders [55–57].

Our study has some notable limitations; first, the lack of a control group where IBD patients would not fast during Ramadan would provide more concrete evidence for our results. Second, a few variables that hypothetically could affect results were not assessed, such as sleep patterns and compliance with medications. The type of diet is also a potential confounder as types of foods may change during Ramadan, especially with an increase in high-calorie foods or overeating, this should be assessed in future studies by a food diary [59, 60]. Third, assessments of disease activity using ultrasound and/or endoscopy were not performed, these could have added confidence to the results. It remains difficult however to get patients to do a colonoscopy twice within one month, it is also difficult logistically for an endoscopy unit to get a large number of patients to have a colonoscopy within one week from the end of Ramadan.

In conclusion, IF for 14 to 15 h during the month of Ramadan did not seem to significantly affect CRP and fCal levels in patients diagnosed with IBD. However, in UC patients, especially older ones, and those with a higher baseline fCal, RIF was associated with a deterioration in their clinical activity indices. Larger studies are required to confirm our initial findings, but for now, it seems prudent to counsel patients diagnosed with IBD that intermittent (Ramadan) fasting is allowed but with caution and vigilance, especially in older UC patients and those with a baseline calprotectin above normal.

## Data Availability

All data generated or analysed during this study are included in this published article. Availability and implementation VCFvariance.pl is a Perl script available at https://github.com/kfletcher88/VCFvariance.
